# Apoptosis antagonizing transcription factor expression and its validation as a potential diagnostic and prognostic biomarker in oral squamous cell carcinoma

**DOI:** 10.3389/fonc.2025.1542730

**Published:** 2025-01-22

**Authors:** Ainiwaerjiang Abudourousuli, Zumulaiti Aierken, Hasiyati Mamuti, Tuxunayi Yimamu, Chengli Da

**Affiliations:** ^1^ Department of Pathology, The First People`s Hospital of Kashi Prefecture, Kashi, China; ^2^ Department of Stomatology, The First People`s Hospital of Kashi Prefecture, Kashi, China; ^3^ Department of Oral and Maxillofacial Surgery, The First People`s Hospital of Kashi Prefecture, Kashi, China

**Keywords:** apoptosis antagonizing transcription factor, oral squamous cell carcinoma, prognostic biomarker, immune microenvironment, expression profile

## Abstract

**Background:**

Oral squamous cell carcinoma (OSCC) is characterized by a high degree of malignancy and poor prognosis. This study aimed to investigate the expression of apoptosis antagonizing transcription factor (AATF) in OSCC, examine its correlation with clinicopathological features, assess its prognostic implications, and explore its potential role in OSCC progression.

**Methods:**

Expression profiles and clinical data of OSCC patients were obtained from The Cancer Genome Atlas (TCGA). Immunohistochemical analysis on tissue microarrays was performed to assess AATF expression in OSCC. Functional enrichment analyses were conducted to identify potential signaling pathways and biological functions associated with AATF. Logistic regression analyses were employed to evaluate the relationship between AATF expression and clinicopathological features. Immune cell infiltration was assessed using single-sample gene set enrichment analysis (ssGSEA). The prognostic value of AATF was determined using Kaplan-Meier and Cox regression analyses. A nomogram was developed to predict overall survival (OS) rates at one, three-, and five years post-cancer diagnosis. Validation of AATF expression was performed using quantitative real-time PCR (qRT-PCR)

**Results:**

AATF was significantly overexpressed in OSCC, and high AATF expression correlated with adverse clinicopathological features, including histologic grade and lymph node metastasis. Functional enrichment analysis revealed several enriched pathways, including epidermis development, immunoglobulin complex, antigen binding and IL-17 signaling pathway. Notably, AATF overexpression was negatively correlated with the infiltration levels of mast cells, interdigitating dendritic cells and Th 17 cells. High AATF expression significantly predicted poorer overall survival (OS) and disease-specific survival (DSS). Multivariate Cox analysis confirmed AATF as an independent negative prognostic marker of OS. Validation via qRT-PCR confirmed the overexpression of AATF in OSCC tissues.

**Conclusion:**

Elevated expression of AATF in OSCC correlates with adverse clinicopathological features and negatively impacts immune cell infiltration. High AATF levels serve as an independent marker of poor OS and DSS. These findings support AATF as a valuable prognostic biomarker and a potential therapeutic target in OSCC, warranting further investigation.

## 1 Background

Oral squamous cell carcinoma (OSCC) is a highly aggressive malignancy of the head and neck, characterized by poor prognosis and high mortality rates (1). Globally, OSCC ranks as the eighth most common cancer and the seventh leading cause of cancer-related death (2). Major risk factors for OSCC include tobacco use, alcohol consumption, poor oral hygiene, and human papillomavirus (HPV) infection (3). Despite significant advances in early diagnosis, surgical interventions, chemotherapy, and immunotherapy, the prognosis for patients with OSCC remains low, with a five-year survival rate of approximately 50% (4). This highlights the critical need for novel diagnostic and prognostic biomarkers (5). Current early diagnostic methods for OSCC largely depend on imaging and histopathological analyses, which have limitations in sensitivity and specificity (6). Therefore, the identification of novel diagnostic and prognostic biomarkers for OSCC is crucial for enhancing patient outcomes and therapeutic effectiveness (7). The AATF located on human chromosome 17, is a member of the ATF/CREB family (7, 8). AATF is a multifunctional protein involved in DNA damage repair, cellular adaptation to hypoxia, oxidative stress response, and the inhibition of apoptosis (9). As a transcriptional coactivator, AATF influences cell cycle progression and apoptosis by regulating key genes such as P53 and P21 (10, 11). Additionally, AATF plays a critical role in chromatin structure and epigenetic regulation through interactions with histone deacetylase 1 (HDAC1), the retinoblastoma protein-E2F transcription factor complex, and specificity protein 1 (SP1) (12). AATF also modulates key signaling pathways, including the p53 tumor suppressor pathway and the NF-κB pathway, to regulate cell survival and proliferation (13). Altered AATF expression has been observed in various cancers, including tumors, colorectal cancer (CRC), lung cancer (LC), and hepatocellular carcinoma (HCC), where its overexpression is associated with poor prognosis (7, 14, 15). Elevated levels of AATF correlate with increased tumor invasiveness and metastasis, as well as decreased patient survival, indicating its potential role in tumorigenesis and progression (16). However, the role of AATF in OSCC remains unexplored, making it essential to investigate its expression and functional mechanisms in this context. This study aims to elucidate the expression and functional mechanisms of AATF in OSCC using bioinformatics and tissue microarray techniques. Our findings indicate that AATF is significantly overexpressed in OSCC and correlates with adverse clinicopathological features, immune cell infiltration, and poor prognosis. We conducted Gene Ontology (GO), Kyoto Encyclopedia of Genes and Genomes (KEGG), and Gene Set Enrichment Analysis (GSEA) to investigate the biological functions and associated signaling pathways of AATF in OSCC. These results offer novel insights into the role of AATF in OSCC and provide a theoretical basis for developing AATF-based diagnostic and therapeutic strategies. In summary, AATF may serve as a potential molecular biomarker for the diagnosis and prognosis assessment of OSCC.

## 2 Materials and methods

### 2.1 Data collection

RNA sequencing (RNA-seq) data and clinical information for patients diagnosed with OSCC were retrieved from The Cancer Genome Atlas (TCGA) database, ensuring the selection of high-quality, curated datasets. The level 3 HTSeq-FPKM format data were normalized to transcripts per million reads (TPM) to facilitate comparative analyses and enhance the robustness of subsequent findings.

### 2.2 Tissue microarray

The tissue microarray utilized in this study was procured from Zhongke Guanghua (Xi’an) Intelligent Biological Co., Ltd. (catalogue number: HN0580c01) ethics approved by same communicated with number: Csya2024029. This array comprised 39 cancerous tissues and 19 control tissues, including adjacent non-cancerous tissues or normal gingival mucosa, enabling a comprehensive assessment of AATF expression across differing tumor environments.

### 2.3 Immunohistochemistry

Tumor and adjacent tissues were fixed in 10% formalin, embedded in paraffin, sectioned into 4–6 μm slices, and mounted onto glass slides. Following deparaffinisation and rehydration, antigen retrieval was achieved via microwave treatment using a citrate buffer (pH 6.0) to enhance antibody binding. The sections were incubated overnight at 4°C with a primary anti-AATF antibody (Bioss, #bs1229R) diluted to 1:200. Subsequently, the sections were treated with a secondary antibody at room temperature for 30 minutes, followed by DAB substrate staining and counterstaining with haematoxylin, providing clear delineation of AATF expression in the tissue architecture.

### 2.4 Differentially expressed gene analysis

Patients with OSCC in the TCGA database were stratified into high and low-expression groups based on the median AATF expression level, ensuring a clear differentiation in biological response. Differentially expressed genes (DEGs) between these groups were identified using the R package DESeq2, applying thresholds of an adjusted p-value < 0.05 and |log2-fold-change (FC)| > 1 for significance. The top 10 DEGs were further assessed for correlation with AATF expression via Spearman correlation analysis, highlighting potential pathways influenced by AATF.

### 2.5 Functional enrichment analysis

Functional enrichment analysis of DEGs, encompassing Gene Ontology (GO) and Kyoto Encyclopedia of Genes and Genomes (KEGG), was conducted using the R package GOplot (version 1.0.2) (Walter et al., 2015). This analysis aimed to elucidate the biological functions and pathways associated with AATF. Gene set enrichment analysis (GSEA) was performed using the R package clusterProfiler, considering an adjusted p-value < 0.05 and a false discovery rate (FDR) < 0.25 as statistically significant for enriched functions or pathways, thereby providing insights into the molecular mechanisms underlying OSCC progression.

### 2.6 Immune infiltration analysis

The levels of immune infiltration for 24 distinct immune cell types were quantified, with relative enrichment scores determined using single-sample gene set enrichment analysis (ssGSEA). To investigate the correlation between AATF expression and immune cell types, Spearman correlation analysis was employed. Differences in immune infiltration levels between high and low AATF expression groups were evaluated using the Wilcoxon rank-sum test, providing insights into the immunological landscape associated with AATF expression in OSCC.

### 2.7 Survival analysis

Survival analysis was conducted utilizing the Kaplan-Meier method alongside the log-rank test, with the cut-off point established at the median AATF expression level. Univariate and multivariate Cox regression analyses were performed to evaluate the impact of clinical variables on patient prognosis. Variables demonstrating a prognostic significance with *p* < 0.1 in the univariate Cox regression analysis were incorporated into the multivariate Cox regression model. The results were visualized using forest plots generated with the R package ggplot2, facilitating a comprehensive interpretation of the survival outcomes.

### 2.8 Statistical analysis

All statistical analyses were executed using R (version 3.6.3). The Wilcoxon rank-sum test and paired t-test were employed to determine the statistical significance of AATF expression across unpaired and paired tissue samples, respectively. Additionally, the Wilcoxon rank-sum test and logistic regression were utilized to explore the correlation between clinical characteristics and AATF expression. All statistical tests were two-sided, with p-values < 0.05 deemed statistically significant, thereby ensuring the robustness of the findings.

### 2.9 Validation

To validate the RNA-seq results and immunohistochemistry findings, quantitative real-time PCR (qRT-PCR) was performed on an independent cohort of 20 OSCC tissue samples and their corresponding adjacent non-cancerous tissues. Total RNA was extracted using the TRIzol reagent (Invitrogen) and reverse-transcribed into cDNA using the PrimeScript RT reagent kit (Takara Bio, Japan). qRT-PCR was conducted using SYBR Green Master Mix (Takara Bio) on a QuantStudio 3 Real-Time PCR System (Applied Biosystems). AATF expression was normalized to GAPDH, and the relative expression levels were calculated using the 2^−ΔΔCt method. Statistical analysis was performed using paired t-tests to compare AATF expression levels between tumors and adjacent tissues.

## 3 Results

### 3.1 AATF is significantly overexpressed in OSCC

**Figure 1:**
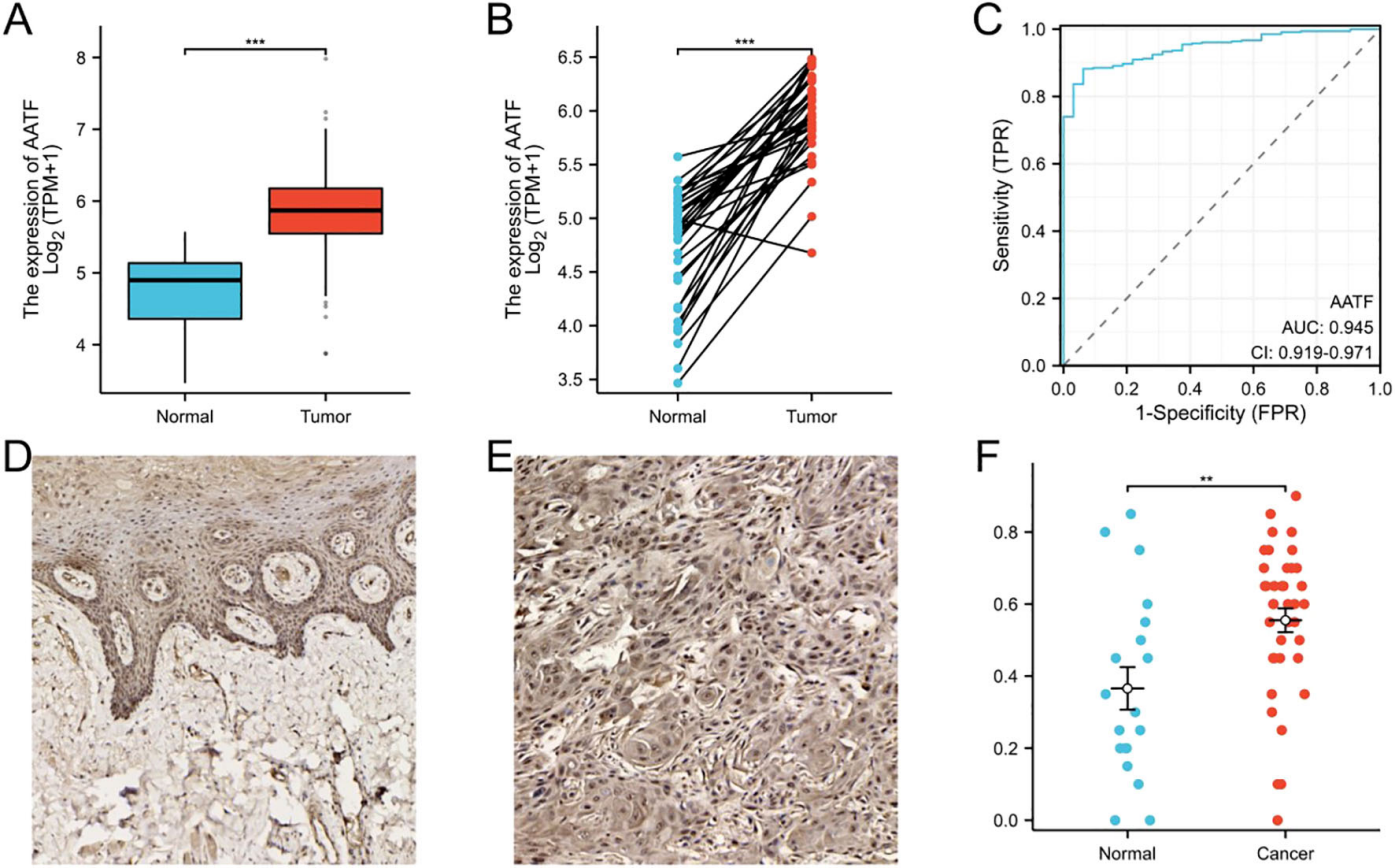
AATF Over Expression in OSCC and Adjacent Non-Cancerous Tissues. **(A)** The expression of AATF in TCGA database. **(B)** The expression of AATF in paired OSCC tissues. **(C)** The ROC curve of AATF. **(D)** Immunohistochemical staining of AATF in adjacent tissues. **(E)** Immunohistochemical staining of AATF in OSCC tissues. **(F)** Quantification of AATF immunohistochemical staining. **: pvalue < 0.01; ***: pvalue < 0.001.

Analysis of data from The Cancer Genome Atlas (TCGA) (http://portal.gdc.cancer.gov/) a comprehensive public database that provides genomic, transcriptomic, and clinical data on various cancer types, revealed a notable upregulation of AATF in OSCC compared to adjacent non-cancerous tissues, as confirmed by the Wilcoxon test (**Figure 1A**). In paired OSCC samples, this differential expression was further corroborated (**Figure 1B**). To assess the diagnostic potential of AATF, a receiver operating characteristic (ROC) curve was generated, yielding an area under the curve (AUC) of 0.945, which indicates high diagnostic accuracy in differentiating OSCC from adjacent tissues (**Figure 1C**). Additionally, tissue microarray analysis and immunohistochemistry were conducted to further validate AATF expression. Immunohistochemical staining demonstrated significantly elevated AATF expression in OSCC compared to adjacent tissues (**Figures 1D, E**). Quantitative analysis revealed a significantly greater staining intensity for AATF in OSCC samples (**Figure 1F**). Collectively, these findings underscore that AATF is markedly overexpressed in OSCC, highlighting its potential as a diagnostic biomarker.

### 3.2 Correlation of AATF expression with clinicopathological features in OSCC

**Figure 2:**
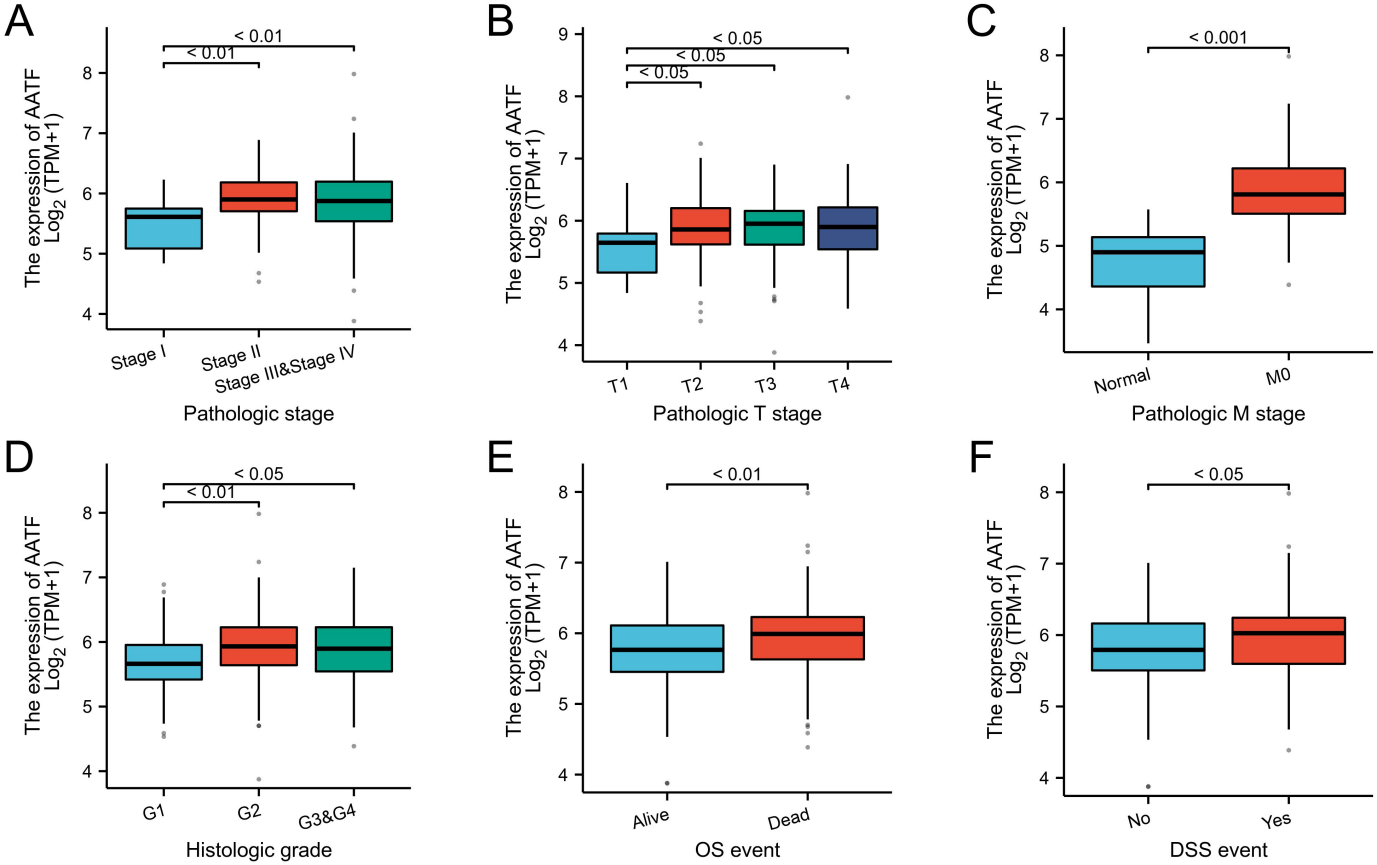
Correlation of AATF Over Expression with Clinicopathological Features in OSCC **(A)** Pathological stage, **(B)**Pathological T stage, **(C)** Pathological M stage, **(D)** Histological grade, **(E)** OS events, **(F)** DSS events.

A comprehensive analysis was conducted using clinical data from 330 OSCC cases sourced from The Cancer Genome Atlas (TCGA) (**Table 1**). Patients were stratified into high-expression (n = 165) and low-expression (n = 165) groups based on the median value of AATF expression. The relationship between AATF expression levels and various clinicopathological features was systematically evaluated. Statistical analyses indicated significant associations between AATF expression and several key pathological parameters, including pathological stage, pathological T stage, pathological M stage, clinical stage, clinical M stage, and histological grade. Notably, AATF expression was significantly correlated with OS (*p* < 0.01) and disease-free survival (*p* < 0.05), as illustrated in **Figures 2A–F**. Additionally, logistic regression analyses identified specific correlations between AATF expression and pathological N stage (*p* = 0.002), clinical stage (*p* = 0.025), and clinical M stage (**Table 2**). These findings underscore the potential of AATF as a significant biomarker associated with adverse clinicopathological features in OSCC, suggesting its relevance in both prognosis and clinical decision-making.

**Table 1:** Clinicopathological characteristics of OSCC patients and AATF expression levels.

Characteristics	Low expression of AATF	High expression of AATF	*P* value
n	165	165	
Age, n (%)			0.958
<= 60	78 (23.7%)	78 (23.7%)	
> 60	86 (26.1%)	87 (26.4%)	
Gender, n (%)			0.153
Female	57 (17.3%)	45 (13.6%)	
Male	108 (32.7%)	120 (36.4%)	
Pathologic stage, n (%)			**0.005**
Stage I	15 (5%)	2 (0.7%)	
Stage II	25 (8.4%)	29 (9.7%)	
Stage III	36 (12%)	25 (8.4%)	
Stage IV	78 (26.1%)	89 (29.8%)	
Pathologic T stage, n (%)			**0.011**
T1	23 (7.5%)	6 (2%)	
T2	51 (16.7%)	49 (16.1%)	
T3	29 (9.5%)	37 (12.1%)	
T4	52 (17%)	58 (19%)	
Pathologic N stage, n (%)			**0.009**
N0	70 (25.4%)	48 (17.4%)	
N1	30 (10.9%)	20 (7.2%)	
N2	43 (15.6%)	63 (22.8%)	
N3	0 (0%)	2 (0.7%)	
Primary therapy outcome, n (%)			0.665
PD	17 (6.1%)	18 (6.5%)	
SD	1 (0.4%)	3 (1.1%)	
PR	1 (0.4%)	2 (0.7%)	
CR	122 (43.9%)	114 (41%)	
Histologic grade, n (%)			**0.008**
G1	36 (11.2%)	16 (5%)	
G2	91 (28.3%)	110 (34.2%)	
G3	31 (9.6%)	36 (11.2%)	
G4	2 (0.6%)	0 (0%)	
Alcohol history, n (%)			0.251
No	57 (17.7%)	48 (14.9%)	
Yes	103 (32%)	114 (35.4%)	
Lymphovascular invasion, n (%)			0.288
No	87 (36.2%)	78 (32.5%)	
Yes	34 (14.2%)	41 (17.1%)	
Lymphnode neck dissection, n (%)			0.630
No	24 (7.3%)	21 (6.4%)	
Yes	140 (42.7%)	143 (43.6%)	
Smoker, n (%)			0.496
No	47 (14.5%)	41 (12.7%)	
Yes	116 (35.8%)	120 (37%)	
Radiation therapy, n (%)			0.882
No	60 (20.3%)	56 (19%)	
Yes	91 (30.8%)	88 (29.8%)	
OS event, n (%)			**< 0.001**
Alive	108 (32.7%)	72 (21.8%)	
Dead	57 (17.3%)	93 (28.2%)	
DSS event, n (%)			**< 0.001**
No	125 (39.9%)	95 (30.4%)	
Yes	33 (10.5%)	60 (19.2%)	
PFI event, n (%)			**0.033**
No	107 (32.4%)	88 (26.7%)	
Yes	58 (17.6%)	77 (23.3%)	

P value < 0.05 is highlighed in bold.

**Table 2:** The logistic regression analysis of AATF in OSCC.

Characteristics	Total (N)	OR (95% CI)	*P* value
Age (> 60 vs. <= 60)	329	1.012 (0.579 – 1.444)	0.958
Gender (Male vs. Female)	330	1.407 (0.938 – 1.877)	0.154
Pathologic stage (Stage III & Stage IV vs. Stage I & Stage II)	299	1.290 (0.754 – 1.826)	0.351
Pathologic T stage (T3&T4 vs. T1&T2)	305	1.578 (1.120 – 2.036)	0.051
Pathologic N stage (N2&N3 vs. N0&N1)	276	2.223 (1.730 – 2.716)	0.002
Primary therapy outcome (PR&CR vs. PD&SD)	278	0.808 (0.130 – 1.487)	0.539
Histologic grade (G3&G4 vs. G1&G2)	322	1.100 (0.567 – 1.632)	0.727
Alcohol history (Yes vs. No)	322	1.314 (0.847 – 1.782)	0.252
Lymphovascular invasion (Yes vs. No)	240	1.345 (0.797 – 1.893)	0.289
Lymphnode neck dissection (Yes vs. No)	328	1.167 (0.537 – 1.798)	0.630
Smoker (Yes vs. No)	324	1.186 (0.695 – 1.676)	0.496
Radiation therapy (Yes vs. No)	295	1.036 (0.569 – 1.504)	0.882

### 3.3 Functional enrichment analysis of AATF-related differentially expressed genes in OSCC

**Figure 3:**
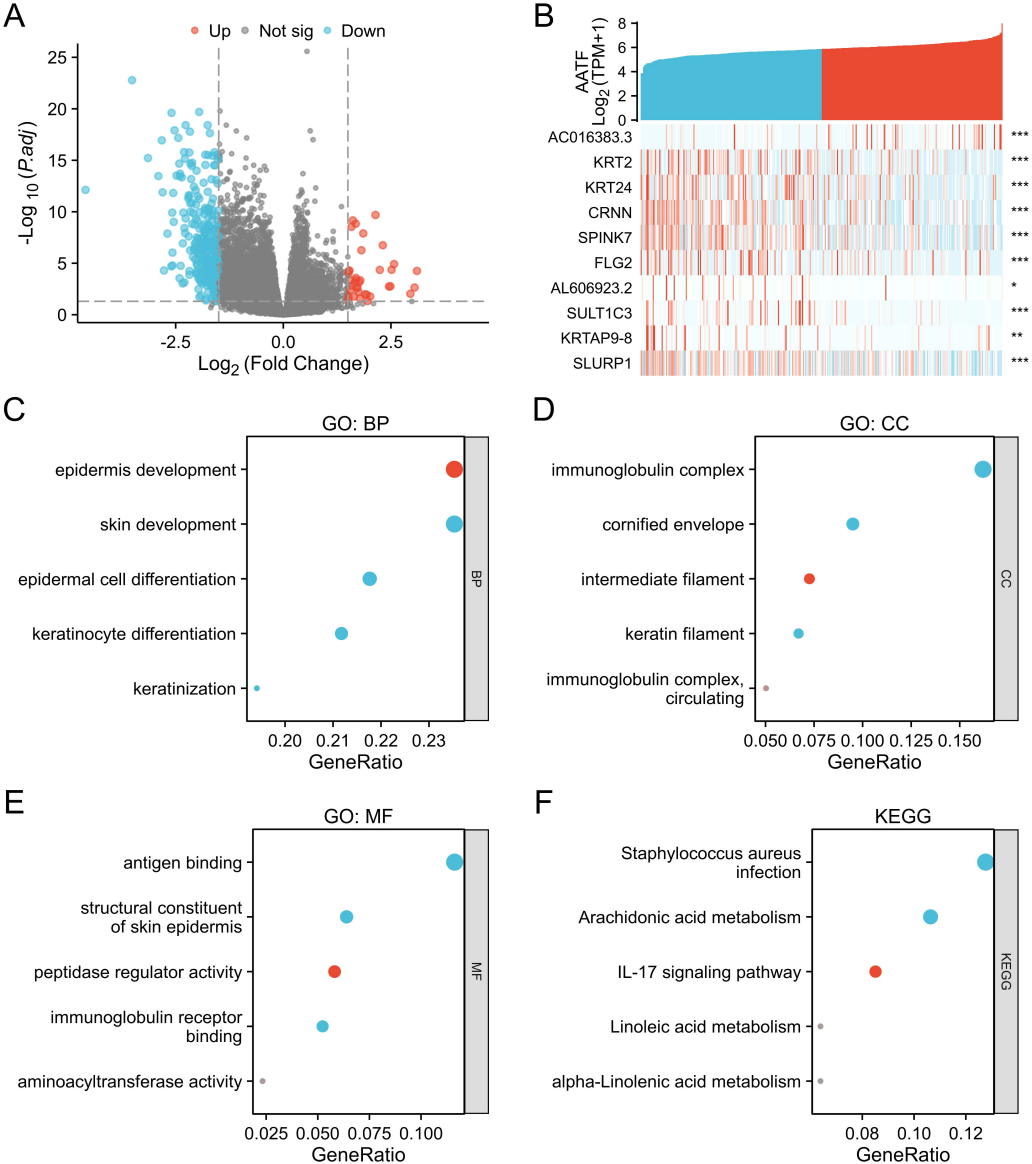
Differentially expressed genes (DEGs) associated with AATF and functional enrichment analysis in OSCC. **(A)** Volcano plot of DEGs (blue and red points indicate significantly downregulated and upregulated DEGs, respectively). **(B)** Heatmap showing the correlation between AATF expression and the top ten DEGs. **(C)** GO biological process (GO-BP) analysis of DEGs. **(D)** GO cellular component (GO-CC) analysis of DEGs. **(E)** GO molecular function (GO-MF) analysis of DEGs. **(F)** KEGG pathway analysis of DEGs. *: pvalue < 0.05; **: pvalue < 0.01; ***: pvalue < 0.001.

A total of 323 differentially expressed genes (DEGs) were identified between the AATF high-expression and low-expression groups, comprising 37 upregulated genes (11.4%) and 286 downregulated genes (88.6%) (adjusted *p*-value < 0.05, |Log^2^-FC| > 1.5) (**Figure 3A**). The relationship between AATF and the top 10 DEGs—AC016383.3, KRT2, FRT24, CRNN, SPINK7, FLG2, AC606923.3, SULT1C3, KRTAP9-8, and SLURP1—is illustrated in **Figure 3B**. Gene Ontology (GO) enrichment analysis revealed significant biological processes (BP), cellular components (CC), and molecular functions (MF) associated with these DEGs. In terms of biological processes, enriched terms included epidermis development, skin development, epidermal cell development, keratinocyte development, and keratinization (**Figure 3C**). For cellular components, the analysis identified significant enrichment in immunoglobulin complexes, cornified envelopes, intermediate filaments, keratin filaments, and circulating immunoglobulin complexes (**Figure 3D**). The molecular function analysis highlighted key activities such as antigen binding, structural constituents of the skin epidermis, peptidase regulator activity, immunoglobulin receptor binding, and aminoacyl transferase activity (**Figure 3E**). Furthermore, KEGG pathway analysis revealed notable pathways, including Staphylococcus aureus infection, arachidonic acid metabolism, IL-17 signaling pathway, linoleic acid metabolism, and alpha-linolenic acid metabolism (**Figure 3F**). These findings underscore the potential biological significance of AATF-related DEGs in the context of OSCC progression and pathology.

### 3.4 GSEA analysis of AATF in OSCC

**Figure 4:**
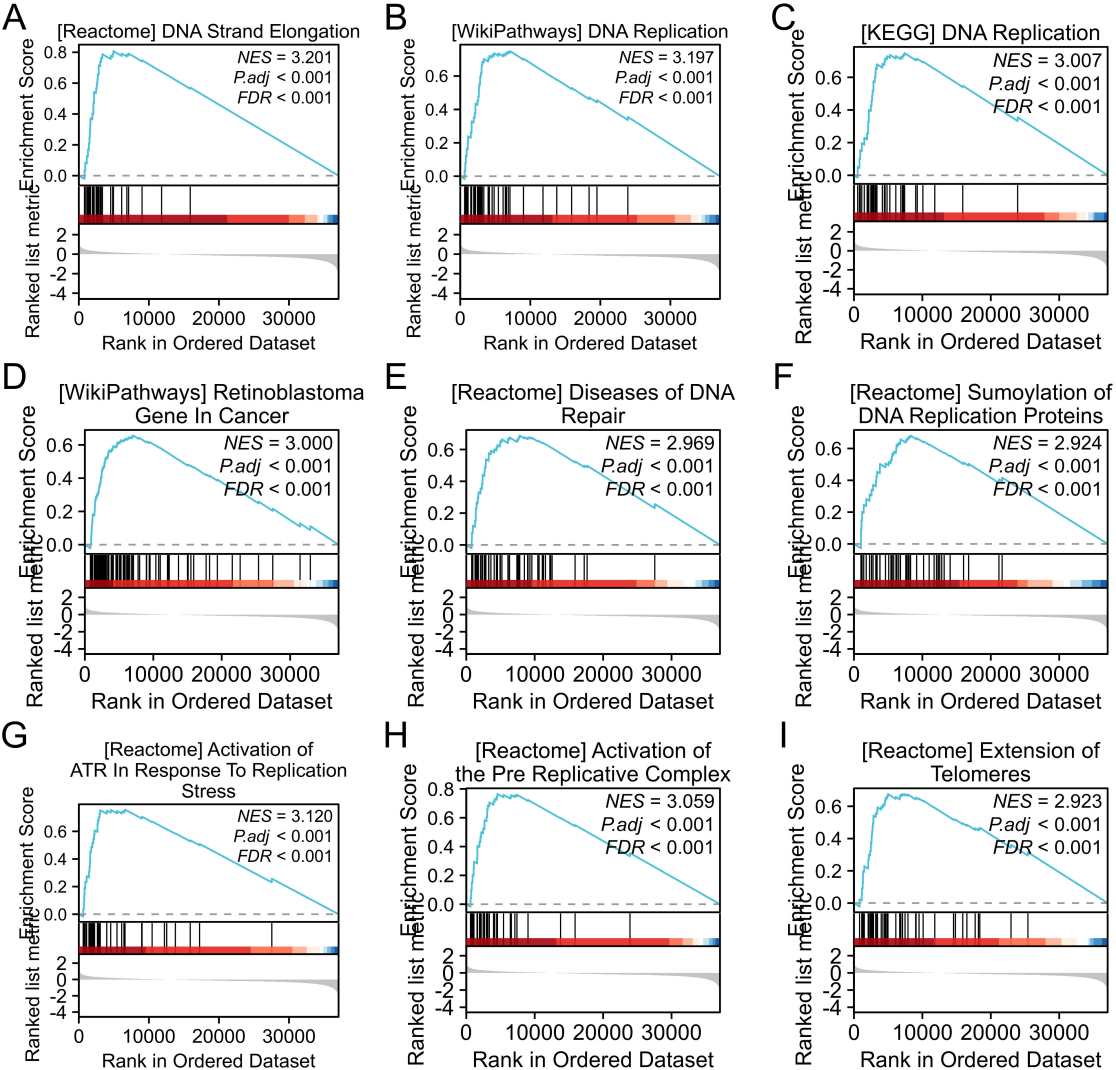
Gene set enrichment analysis (GSEA) of differentially expressed gene sets. **(A)** DNA strand elongation; **(B)** DNA replication; **(C)** KEGG DNA replication pathway; **(D)** GO molecular function of gene sets; **(E)** DNA repair-related diseases; **(F)** SUMOylation of DNA replication proteins; **(G)** Activation of alternative end-joining (ART) in response to replication stress; **(H)** Pre-replication complex activation; **(I)** Telomerase elongation.

To elucidate the biological implications of AATF-related differentially expressed genes (DEGs) in oral squamous cell carcinoma (OSCC), we performed a Gene Set Enrichment Analysis (GSEA). This analysis revealed significant enrichments in key biological processes and pathways associated with DNA metabolism and cellular proliferation. Notably, GSEA indicated enrichments in DNA strand elongation and DNA replication processes, suggesting a crucial role for AATF in facilitating the replication machinery essential for cancer cell growth (**Figures 4A–I**). The KEGG pathway analysis further underscored the importance of the DNA replication pathway in OSCC. We also observed significant associations with retinoblastoma (RB) genes in cancer, implicating AATF in the regulation of cell cycle checkpoints. Enrichment in DNA repair disorders emphasized AATF’s potential role in maintaining genomic stability. Additionally, the analysis highlighted SUMOylation of DNA replication proteins, indicating a sophisticated regulatory mechanism by AATF. The activation of alternative end-joining (ART) pathways in response to replication stress points to AATF’s involvement in genomic instability. Furthermore, GSEA indicated the activation of pre-replication complexes and extended telomerase activity, suggesting AATF’s influence on telomere maintenance, which is critical for sustaining cancer cell proliferation. These findings collectively highlight AATF’s multifaceted roles in regulating DNA-related processes in OSCC.

### 3.5 Correlation of AATF with immune infiltration

**Figure 5:**
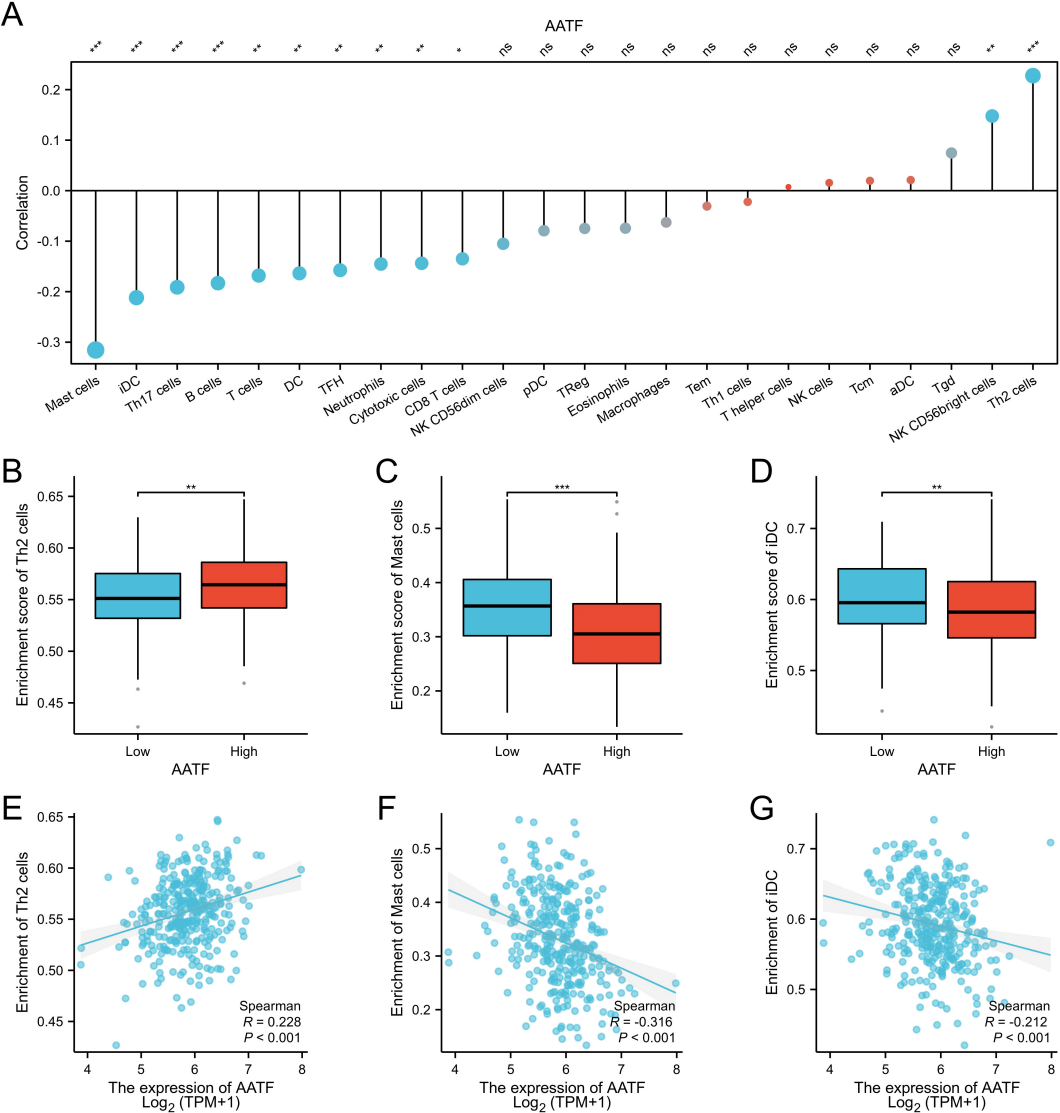
Correlation between AATF Expression and Immune Cell Infiltration in OSCC **(A)** Correlation between AATF expression and various immune cell types. **(B)** Effect of AATF expression on TH2 cell infiltration. **(C)** Impact of AATF expression on mast cell infiltration. **(D)** Association between AATF and iDC cell infiltration. **(E)** Correlation between AATF and TH2 cells. **(F)** Correlation between AATF and mast cells. **(G)** Association between AATF expression and iDC cells. *: pvalue < 0.05; **: pvalue < 0.01; ***: pvalue < 0.001. ns, not significant.

To investigate the relationship between AATF expression and immune cell infiltration in oral squamous cell carcinoma (OSCC), we employed single-sample gene set enrichment analysis (ssGSEA) (**Figure 5A** **).** The analysis revealed a complex correlation pattern between AATF levels and various immune cell types. Specifically, AATF expression exhibited a negative correlation with mast cells, immature dendritic cells (iDCs), and TH17 cells (**Figures 5C, D, F, G**), suggesting that elevated AATF levels may suppress the infiltration of these immune cells, which are crucial for anti-tumor immunity. Conversely, AATF expression was positively correlated with Th2 cells (**Figures 5B, E**), indicating that high AATF levels may facilitate the recruitment or activation of these immune cell populations, which can be associated with tumor progression. These findings imply that AATF may play a pivotal role in modulating the immune microenvironment in OSCC, potentially influencing disease outcomes through its regulatory effects on immune cell infiltration. Further investigation into these dynamics may elucidate AATF’s potential as a therapeutic target for enhancing anti-tumour immunity in OSCC.

### 3.6 AATF expression and prognosis

**Figure 6:**
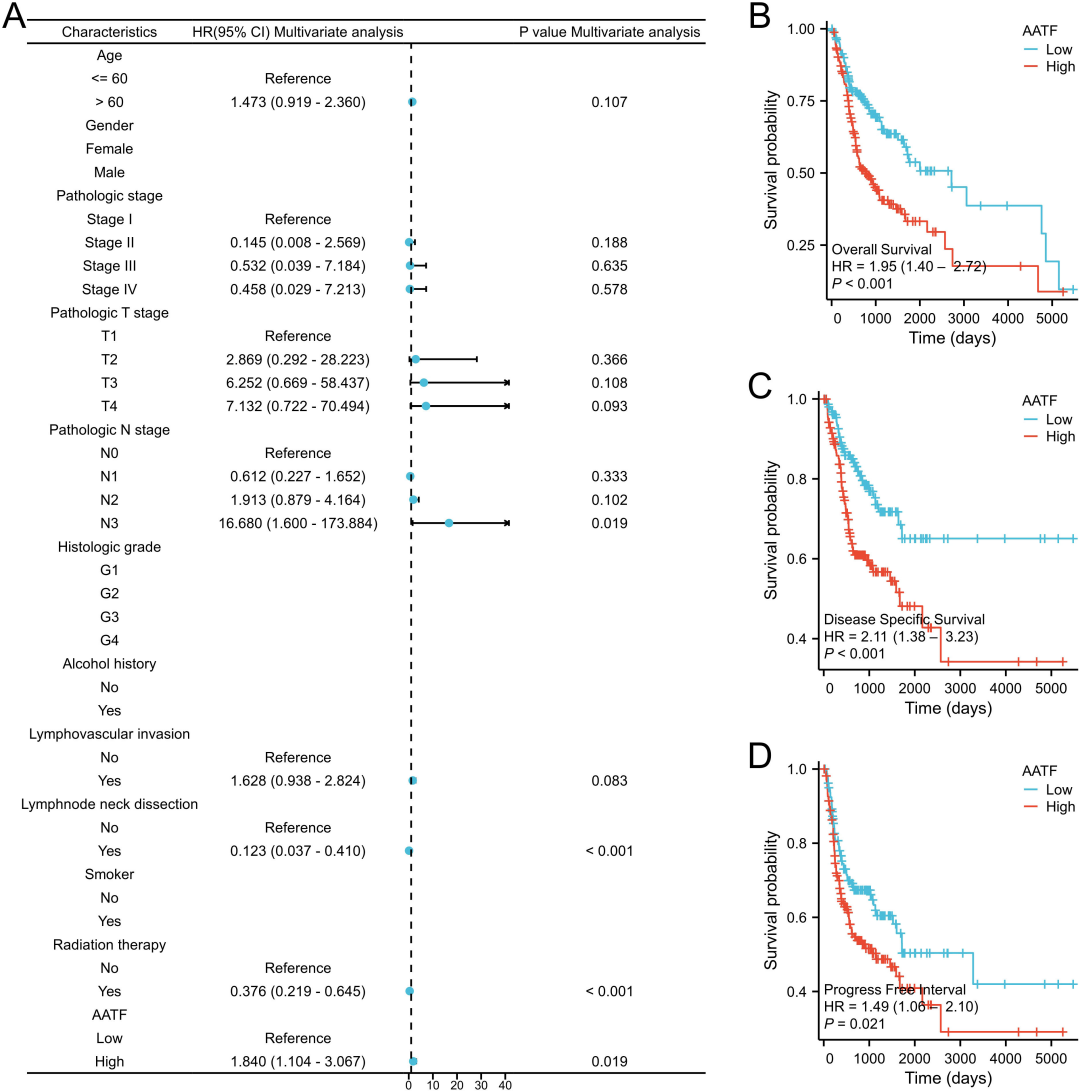
Association of high AATF expression with poor prognosis in OSCC patients. **(A)** Multivariate Cox analysis; **(B)** Overall survival (OS); **(C)** Disease-specific survival (DSS); **(D)** Progression-free interval (PFI).

The relationship between AATF expression and patient prognosis was thoroughly evaluated through the analysis of OS, disease-specific survival (DSS), and progression-free interval (PFI) in patients with OSCC, employing the Kaplan-Meier method. The results demonstrated a significant correlation between high AATF expression levels and poor OS outcomes (P < 0.001, **Figures 6A–D**). Specifically, patients exhibiting elevated AATF levels were found to have markedly worse prognoses, with significantly reduced DSS (P < 0.001) and PFI (P = 0.012). These findings indicate that increased expression of AATF is associated with adverse survival outcomes in OSCC patients, highlighting its potential role as a prognostic biomarker.

### 3.7 Construction and validation of a prognostic nomogram

**Figure 7:**
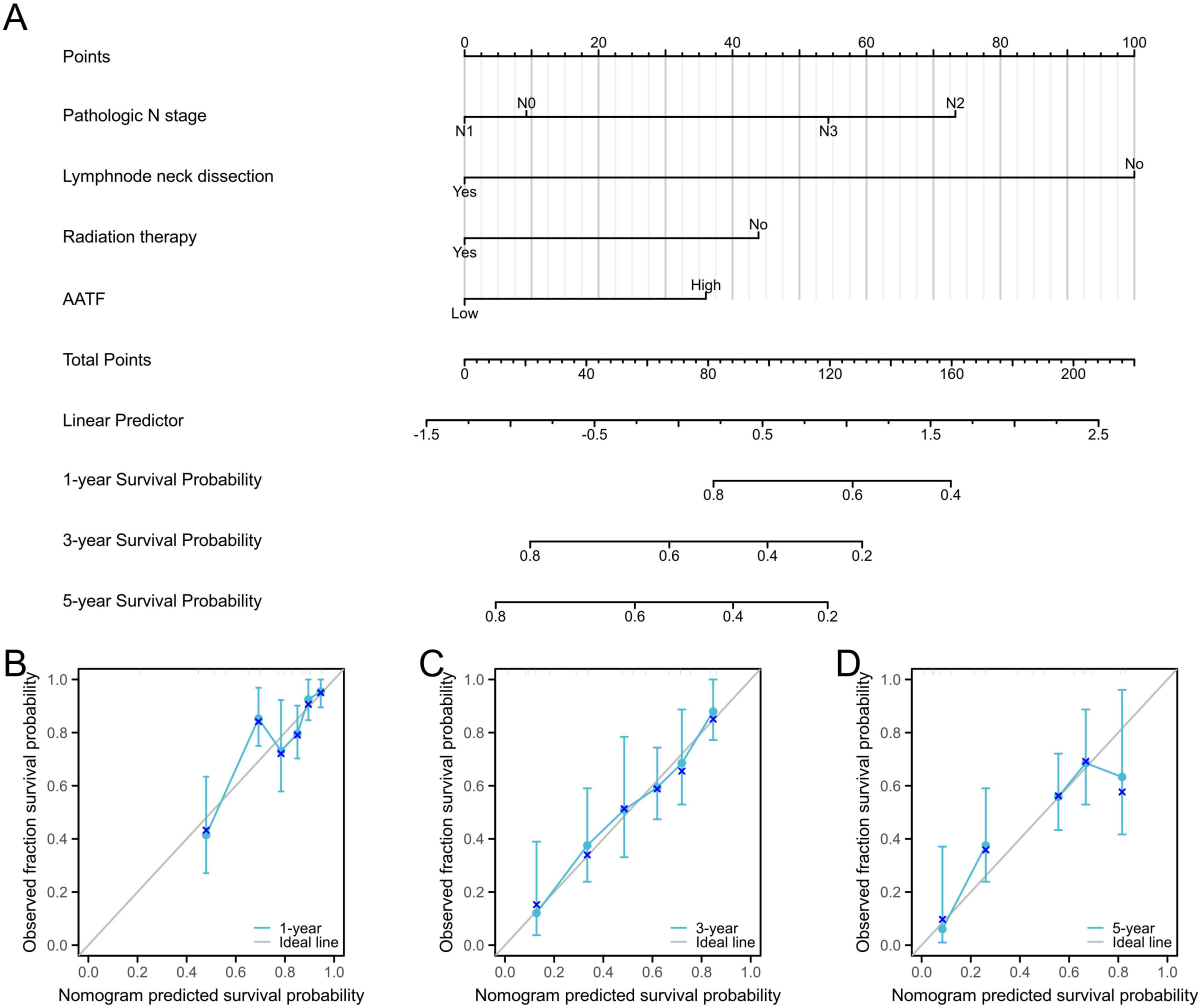
Prognostic diagnostic plots for predicting 1-year, 3-year, and 5-year overall survival in OSCC patients. **(A)** Prognostic diagnostic plot developed for predicting 1-year, 3-year, and 5-year overall survival. **(B-D)** Calibration curves for 1-year, 3-year, and 5-year overall survival predictions in OSCC patients.

To improve prognostic prediction for OSCC patients, we developed a prognostic nomogram that incorporates independent predictors of OS. This nomogram (**Figure 7A**) serves as a visual tool, where higher scores correspond to a poorer prognosis, allowing for personalized risk assessment. The predictive accuracy of the nomogram was rigorously evaluated using calibration curves (**Figures 7B–D**), which demonstrated its reliability in estimating survival probabilities. The bootstrap resampling consistency index (C-index) for the nomogram was calculated at 0.718 (95% Confidence Interval [CI]: 0.692-0.744), indicating a moderate level of accuracy in predicting OS among OSCC patients. This nomogram may thus serve as a valuable resource for clinicians in making informed treatment decisions and tailoring patient management strategies.

### 3.8 qRT-PCR analysis

The qRT-PCR analysis confirmed the overexpression of AATF in OSCC tissues compared to adjacent normal tissues, consistent with the RNA-seq and immunohistochemistry findings. AATF expression was significantly higher in tumor tissues (mean ± SD: 3.62 ± 1.21) compared to adjacent tissues (mean ± SD: 1.18 ± 0.54, p < 0.001). Moreover, the qRT-PCR results demonstrated a positive correlation (r = 0.84, p < 0.001) with the RNA-seq-derived AATF expression levels, reinforcing the reliability of the transcriptomic data.

## 4 Discussion

The global incidence of OSCC has been escalating, particularly in China (17), where distinct epidemiological trends are emerging, with an anticipated 30% annual increase in incidence by 2030 (18). Recent statistics indicate a concerning rise in the annual incidence rate of OSCC in this region, accompanied by a troubling trend toward younger age at onset (19). The high recurrence rates and relatively low five-year survival rates for OSCC underscore the urgent need for enhanced early diagnostic and treatment strategies. Current diagnostic methods and therapeutic approaches often fall short of improving patient outcomes (20). Given the invasive nature and recurrence propensity associated with OSCC, the identification of novel diagnostic and prognostic biomarkers is critical for advancing patient care and treatment efficacy. Recent studies have highlighted several potential biomarkers, including those related to genetic mutations, epigenetic changes, and protein expression profiles, which can provide invaluable insights for early diagnosis, treatment planning, and prognosis assessment in OSCC. For instance, biomarkers such as p16INK4a, EGFR, and PD-L1 have been extensively studied, with varying levels of success in predicting patient outcomes and treatment responses (21). However, these biomarkers often lack the sensitivity and specificity needed for widespread clinical application. In contrast, our findings suggest that AATF may serve as a promising molecular marker.

As a candidate oncogene, AATF plays a pivotal regulatory role in various malignancies (22). However, its specific contributions and clinical significance in OSCC have not been fully elucidated. Our study, leveraging analyses from the TCGA database and OSCC tissue microarrays, revealed significant overexpression of AATF in OSCC. This overexpression correlates closely with adverse clinicopathological features, including histological type, pathological T stage, M stage, and clinical stage. These findings suggest a potential association between AATF and the development of OSCC. The oncogenic effects of AATF may be mediated through its influence on multiple cancer-related signaling pathways, such as the modulation of c-Myc translation, the IL-6/IL-6R signaling axis, and the Src/p190B pathway (23, 24). Prior research has demonstrated that high AATF expression can lead to the generation of reactive oxygen species (ROS), which amplify YY-1/EGFR/MnSOD signaling and enhance cancer cell invasion in lung cancer (25, 26). However, these findings do not fully clarify AATF’s role in OSCC, necessitating further investigation into its biological functions and signaling pathways within this context.

To explore the regulatory pathways linked to aberrant AATF expression in OSCC, we conducted differential expression analysis using the TCGA database. Gene Ontology (GO) and Kyoto Encyclopedia of Genes and Genomes (KEGG) analyses highlighted critical pathways associated with AATF in OSCC, including those involved in DNA replication, pigment metabolism, retinoid and cholesterol metabolism, and lipid digestion and absorption. Gene Set Enrichment Analysis (GSEA) further underscored a correlation between AATF expression and DNA replication. These findings suggest that increased AATF expression may facilitate OSCC carcinogenesis by modulating cancer cell metabolism and enhancing DNA replication. The significance of tumor immunotherapy is increasingly acknowledged, with treatment efficacy closely tied to the degree of immune cell infiltration within tumors. Elevated immune cell infiltration often correlates with improved responses to immunotherapy (27, 28). Utilizing single-sample gene set enrichment analysis (ssGSEA) scores derived from the TCGA cohort, we found that high AATF expression negatively correlated with eosinophils, NK CD56bright cells, and TH17 cells, while showing a positive correlation with TH2 cells, macrophages, and Th1 cells. These observations suggest that elevated AATF expression may play a crucial role in the immune evasion mechanisms employed by OSCC cells. Prior studies have established an association between AATF expression and decreased survival rates across various cancers (29, 30). This study aimed to assess the prognostic utility of AATF in OSCC, thereby evaluating its potential as a prognostic marker. Our findings indicate that aberrant AATF expression correlates with overall survival (OS), disease-specific survival (DSS), and progression-free interval (PFI) in OSCC patients, positioning AATF as a promising therapeutic target.

The validation using qRT-PCR provided robust evidence supporting the overexpression of AATF in OSCC tissues, aligning with both RNA-seq and immunohistochemical findings. The concordance between these methods underscores the reliability of AATF as a potential biomarker for OSCC. The RNA-seq analysis revealed significant DEGs associated with AATF, implicating its involvement in tumorigenesis. Functional enrichment analysis highlighted pathways related to cell cycle regulation and immune modulation, suggesting AATF’s multifaceted role in OSCC progression. Consistent with these findings, qRT-PCR demonstrated significantly elevated AATF levels, confirming its overexpression in tumor tissues. Immunohistochemistry findings provided spatial insights into AATF expression, showing predominant localization within tumor cells compared to adjacent non-cancerous tissues. The strong positive correlation between AATF expression and immune infiltration of specific cell types suggests its potential role in modulating the tumor microenvironment (31). The qRT-PCR validation reinforced these observations, indicating that AATF could influence immune dynamics in OSCC. The survival analysis further emphasized the prognostic value of AATF, with high expression levels correlating with poorer patient outcomes. This relationship highlights the potential utility of AATF not only as a biomarker but also as a therapeutic target. The integration of RNA-seq, immunohistochemistry, and qRT-PCR results strengthens the evidence base for AATF’s role in OSCC.

Despite these valuable insights, this study has inherent limitations. Our findings primarily stem from analyses of public database data and immunohistochemical validation using tissue microarrays, lacking validation through large-scale clinical samples. Furthermore, while we have identified potential mechanisms underlying AATF’s role in OSCC, additional experimental studies are necessary to validate the specific molecular pathways involved.

## 5 Conclusion

Our study highlights that AATF is significantly overexpressed in OSCC and correlates with adverse clinicopathological features and poor prognosis. AATF may play a critical role in OSCC development through various signaling pathways and immune regulatory mechanisms. These findings provide new perspectives on AATF’s involvement in OSCC and offer a theoretical framework for the development of AATF-based diagnostic and therapeutic strategies. Given its potential as a key regulator in OSCC progression, future research should focus on further elucidating the precise molecular mechanisms by which AATF contributes to tumorigenesis and metastasis. Moreover, exploring the therapeutic targeting of AATF, either alone or in combination with existing treatment modalities, could open new avenues for improving treatment outcomes in OSCC patients.

## Data Availability

The original contributions presented in the study are included in the article/supplementary material. Further inquiries can be directed to the corresponding author.
